# 4,4′-Dimethoxy­benzophenone: a triclinic polymorph

**DOI:** 10.1107/S1600536808017315

**Published:** 2008-06-13

**Authors:** Hoong-Kun Fun, S. Franklin, Samuel Robinson Jebas, T. Balasubramanian

**Affiliations:** a X-ray Crystallography Unit, School of Physics, Universiti Sains Malaysia, 11800 USM, Penang, Malaysia; b Department of Physics, National Institute of Technology, Tiruchirappalli 620 015, India

## Abstract

The title compound, C_15_H_14_O_3_, has been found to crystallize as a new triclinic polymorph. The asymmetric unit of the present structure, as in the previously reported monoclinic structure [Norment & Karle (1962 ▶). *Acta Cryst*. **15**, 873–878], contains two independent mol­ecules, which differ slightly in the orientations of the two benzene rings. The crystal packing of the triclinic polymorph is stabilized by inter­molecular C—H⋯O hydrogen bonds and C—H⋯π inter­actions.

## Related literature

For the monoclinic polymorph of 4,4′-dimethoxy­benzo­phenone, see: Norment & Karle (1962 ▶). For bond-length data, see: Allen *et al.* (1987 ▶).
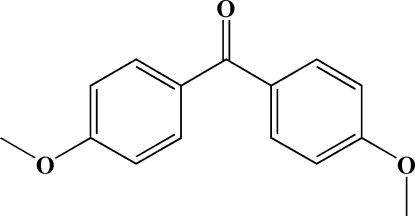


## Experimental

### 

#### Crystal data

C_15_H_14_O_3_
*M* *_r_* = 242.26
Triclinic, 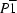

*a* = 9.4296 (2) Å
*b* = 9.4569 (2) Å
*c* = 14.7963 (3) Å
α = 76.945 (1)°
β = 78.813 (1)°
γ = 70.670 (1)°
*V* = 1202.65 (4) Å^3^
*Z* = 4
Mo *K*α radiation
μ = 0.09 mm^−1^
*T* = 100.0 (1) K
0.50 × 0.19 × 0.16 mm

#### Data collection

Bruker SMART APEXII CCD area-detector diffractometer
Absorption correction: multi-scan (*SADABS*; Bruker, 2005 ▶) *T*_min_ = 0.955, *T*_max_ = 0.985
26162 measured reflections
6478 independent reflections
4651 reflections with *I* > 2σ(*I*))
*R*_int_ = 0.035

#### Refinement

*R*[*F* ^2^ > 2σ(*F* ^2^)] = 0.059
*wR*(*F* ^2^) = 0.170
*S* = 1.09
6478 reflections
329 parameters
H-atom parameters constrained
Δρ_max_ = 0.67 e Å^−3^
Δρ_min_ = −0.24 e Å^−3^

### 

Data collection: *APEX2* (Bruker, 2005 ▶); cell refinement: *APEX2*; data reduction: *SAINT* (Bruker, 2005 ▶); program(s) used to solve structure: *SHELXTL* (Sheldrick, 2008 ▶); program(s) used to refine structure: *SHELXTL*; molecular graphics: *SHELXTL*; software used to prepare material for publication: *SHELXTL* and *PLATON* (Spek, 2003 ▶).

## Supplementary Material

Crystal structure: contains datablocks global, I. DOI: 10.1107/S1600536808017315/ci2608sup1.cif

Structure factors: contains datablocks I. DOI: 10.1107/S1600536808017315/ci2608Isup2.hkl

Additional supplementary materials:  crystallographic information; 3D view; checkCIF report

## Figures and Tables

**Table 1:** Hydrogen-bond geometry (Å, °)

*D*—H⋯*A*	*D*—H	H⋯*A*	*D*⋯*A*	*D*—H⋯*A*
C14*B*—H14*E*⋯O1*B*^i^	0.96	2.59	3.446 (2)	149
C9*B*—H9*B*⋯*Cg*1^ii^	0.93	2.84	3.5252 (17)	132
C12*B*—H12*B*⋯*Cg*1^iii^	0.93	2.78	3.5223 (16)	137
C4*B*—H4*B*⋯*Cg*2^iv^	0.93	2.88	3.6301 (18)	138
C9*A*—H9*A*⋯*Cg*3^iii^	0.93	2.92	3.5723 (16)	128
C12*A*—H12*A*⋯*Cg*3^ii^	0.93	2.88	3.5651 (16)	132
C4*A*—H4*A*⋯*Cg*4^v^	0.93	2.90	3.6376 (17)	138

Symmetry codes: (i) 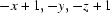; (ii) 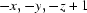; (iii) 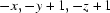; (iv) 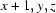; (v) 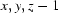. *Cg*1, *Cg*2, *Cg*3 and *Cg*4 are the centroids of the C1*A*–C6*A*, C8*A*–C13*A*, C1*B*–C13*B* and C8*B*–C13*B* rings, respectively.

**Fig. 1.**
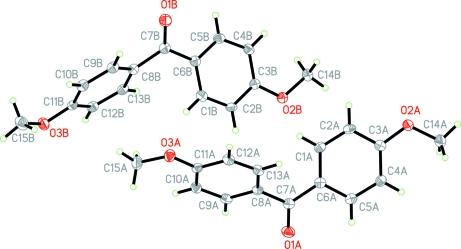
The asymmetric unit of the title compound, showing 50% probability displacement ellipsoids and the atom-numbering scheme.

**Fig. 2.**
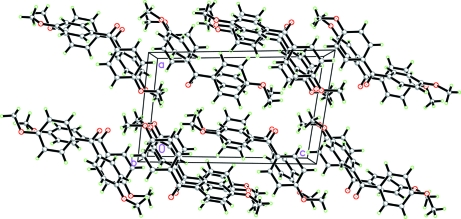
The crystal packing of the title compound, viewed along the b axis. Hydrogen bonds are shown as dashed lines.

## supplementary crystallographic information

### Comment

The crystal structure of the title compound has previously been reported in the monoclinic space group P2_1_/a (Norment & Karle, 1962). We report here the structure of a second polymorph which crystallizes in the triclinic space group P1.

The asymmetric unit of the triclinic polymporph contains two crystallographically independent molecules (Fig.1), similar to the monoclinic form. Bond lengths and angles of the molecules agree with each other and show normal values (Allen *et al.*, 1987). The two independent molecules differ slightly in the orientations of the two benzene rings. The dihedral angle formed by C1A-C6A and C8A-C13A rings is 52.12 (8)° and that between C1B-C6B and C8B-C13B planes is 55.73 (7)°. These dihedral angles are comparable to those observed in the monoclinic polymorph.

The crystal packing is stabilized by intermolecular C—H···O hydrogen bonds (Fig.2) and C—H···π interactions.

### Experimental

The title compound was purchased from Merck and single crystals suitable for X-ray diffraction studies were obtained by slow evaporation of an ethanol solution.

### Refinement

H atoms were positioned geometrically [C-H = 0.93 Å (aromatic) and 0.96 Å (methyl)] and refined using a riding model, with *U*_iso_(H) = 1.2*U*_eq_(C) and 1.5*U*_eq_(methyl C). A rotating group model was used for the methyl group.

### Crystal data

**Table:**

C_15_H_14_O_3_	*Z* = 4
*M**_r_* = 242.26	*F*_000_ = 512
Triclinic, *P*1	*D*_x_ = 1.338 Mg m^−^^3^
Hall symbol: -P 1	Mo *K*α radiation λ = 0.71073 Å
*a* = 9.4296 (2) Å	Cell parameters from 5758 reflections
*b* = 9.4569 (2) Å	θ = 2.3–28.8º
*c* = 14.7963 (3) Å	µ = 0.09 mm^−^^1^
α = 76.945 (1)º	*T* = 100.0 (1) K
β = 78.813 (1)º	Needle, colourless
γ = 70.670 (1)º	0.50 × 0.19 × 0.16 mm
*V* = 1202.65 (4) Å^3^	

### Data collection

**Table:**

Bruker SMART APEXII CCD area-detector diffractometer	6478 independent reflections
Radiation source: fine-focus sealed tube	4651 reflections with *I* > 2σ(*I*))
Monochromator: graphite	*R*_int_ = 0.035
*T* = 100.0(1) K	θ_max_ = 29.3º
φ and ω scans	θ_min_ = 2.3º
Absorption correction: multi-scan(SADABS; Bruker, 2005)	*h* = −12→11
*T*_min_ = 0.955, *T*_max_ = 0.985	*k* = −12→12
26162 measured reflections	*l* = −20→20

### Refinement

**Table:**

Refinement on *F*^2^	Secondary atom site location: difference Fourier map
Least-squares matrix: full	Hydrogen site location: inferred from neighbouring sites
*R*[*F*^2^ > 2σ(*F*^2^)] = 0.059	H-atom parameters constrained
*wR*(*F*^2^) = 0.170	*w* = 1/[σ^2^(*F*_o_^2^) + (0.0952*P*)^2^ + 0.106*P*] where *P* = (*F*_o_^2^ + 2*F*_c_^2^)/3
*S* = 1.09	(Δ/σ)_max_ = 0.001
6478 reflections	Δρ_max_ = 0.67 e Å^−^^3^
329 parameters	Δρ_min_ = −0.24 e Å^−^^3^
Primary atom site location: structure-invariant direct methods	Extinction correction: none

### Special details

**Table:**

Experimental. The data was collected with the Oxford Cyrosystem Cobra low-temperature attachment.
Geometry. All e.s.d.'s (except the e.s.d. in the dihedral angle between two l.s. planes) are estimated using the full covariance matrix. The cell e.s.d.'s are taken into account individually in the estimation of e.s.d.'s in distances, angles and torsion angles; correlations between e.s.d.'s in cell parameters are only used when they are defined by crystal symmetry. An approximate (isotropic) treatment of cell e.s.d.'s is used for estimating e.s.d.'s involving l.s. planes.
Refinement. Refinement of *F*^2^ against ALL reflections. The weighted *R*-factor *wR* and goodness of fit *S* are based on *F*^2^, conventional *R*-factors *R* are based on *F*, with *F* set to zero for negative *F*^2^. The threshold expression of *F*^2^ > σ(*F*^2^) is used only for calculating *R*-factors(gt) *etc*. and is not relevant to the choice of reflections for refinement. *R*-factors based on *F*^2^ are statistically about twice as large as those based on *F*, and *R*- factors based on ALL data will be even larger.

### Fractional atomic coordinates and isotropic or equivalent isotropic displacement parameters (Å^2^)

**Table:**

	*x*	*y*	*z*	*U*_iso_*/*U*_eq_	
O1A	−0.28278 (12)	0.49910 (12)	0.23294 (8)	0.0249 (3)	
O2A	0.34179 (12)	0.13120 (12)	0.02987 (7)	0.0214 (3)	
O3A	−0.28335 (13)	0.14327 (12)	0.64984 (7)	0.0229 (3)	
C1A	0.08731 (17)	0.22148 (16)	0.23864 (10)	0.0184 (3)	
H1A	0.0909	0.1987	0.3028	0.022*	
C2A	0.21523 (18)	0.16537 (16)	0.17906 (11)	0.0194 (3)	
H2A	0.3048	0.1064	0.2030	0.023*	
C3A	0.21106 (17)	0.19668 (15)	0.08281 (10)	0.0178 (3)	
C4A	0.07749 (17)	0.28874 (16)	0.04661 (10)	0.0191 (3)	
H4A	0.0742	0.3113	−0.0176	0.023*	
C5A	−0.04971 (18)	0.34589 (16)	0.10750 (10)	0.0193 (3)	
H5A	−0.1382	0.4080	0.0834	0.023*	
C6A	−0.04823 (17)	0.31234 (15)	0.20434 (10)	0.0174 (3)	
C7A	−0.18690 (17)	0.38447 (16)	0.26527 (10)	0.0183 (3)	
C8A	−0.20800 (16)	0.31981 (16)	0.36677 (10)	0.0173 (3)	
C9A	−0.28139 (17)	0.41828 (16)	0.43012 (11)	0.0191 (3)	
H9A	−0.3135	0.5225	0.4081	0.023*	
C10A	−0.30776 (17)	0.36486 (16)	0.52506 (10)	0.0196 (3)	
H10A	−0.3546	0.4328	0.5663	0.024*	
C11A	−0.26370 (17)	0.20849 (16)	0.55851 (10)	0.0176 (3)	
C12A	−0.19281 (17)	0.10750 (16)	0.49558 (11)	0.0193 (3)	
H12A	−0.1654	0.0031	0.5172	0.023*	
C13A	−0.16374 (17)	0.16278 (16)	0.40171 (10)	0.0182 (3)	
H13A	−0.1140	0.0950	0.3607	0.022*	
C14A	0.3446 (2)	0.1650 (2)	−0.07020 (11)	0.0283 (4)	
H14A	0.4420	0.1114	−0.0991	0.042*	
H14B	0.3261	0.2725	−0.0914	0.042*	
H14C	0.2675	0.1338	−0.0869	0.042*	
C15A	−0.3511 (2)	0.24333 (18)	0.71693 (11)	0.0272 (4)	
H15A	−0.3531	0.1843	0.7789	0.041*	
H15B	−0.4528	0.3008	0.7051	0.041*	
H15C	−0.2928	0.3117	0.7115	0.041*	
O1B	0.29024 (13)	−0.00749 (12)	0.75911 (8)	0.0274 (3)	
O2B	0.28485 (12)	0.35935 (12)	0.34377 (7)	0.0210 (3)	
O3B	−0.31914 (13)	0.37626 (12)	0.96641 (7)	0.0235 (3)	
C1B	0.09869 (17)	0.26402 (16)	0.57589 (11)	0.0186 (3)	
H1B	−0.0001	0.2827	0.6062	0.022*	
C2B	0.12419 (17)	0.32062 (16)	0.48158 (10)	0.0182 (3)	
H2B	0.0426	0.3759	0.4487	0.022*	
C3B	0.27200 (17)	0.29506 (15)	0.43542 (10)	0.0167 (3)	
C4B	0.39422 (17)	0.20963 (16)	0.48461 (10)	0.0189 (3)	
H4B	0.4930	0.1920	0.4544	0.023*	
C5B	0.36678 (17)	0.15134 (16)	0.57903 (10)	0.0189 (3)	
H5B	0.4481	0.0927	0.6113	0.023*	
C6B	0.21978 (17)	0.17880 (15)	0.62655 (10)	0.0177 (3)	
C7B	0.19627 (17)	0.10850 (16)	0.72667 (10)	0.0189 (3)	
C8B	0.05613 (17)	0.17899 (16)	0.78730 (10)	0.0182 (3)	
C9B	−0.00698 (18)	0.08532 (16)	0.85877 (10)	0.0199 (3)	
H9B	0.0361	−0.0197	0.8657	0.024*	
C10B	−0.13333 (18)	0.14621 (17)	0.92001 (11)	0.0205 (3)	
H10B	−0.1757	0.0824	0.9667	0.025*	
C11B	−0.19608 (17)	0.30392 (16)	0.91082 (10)	0.0184 (3)	
C12B	−0.13197 (18)	0.39894 (16)	0.84036 (10)	0.0192 (3)	
H12B	−0.1723	0.5040	0.8351	0.023*	
C13B	−0.00913 (17)	0.33727 (16)	0.77866 (10)	0.0183 (3)	
H13B	0.0310	0.4012	0.7308	0.022*	
C14B	0.43412 (18)	0.33423 (19)	0.29271 (11)	0.0255 (4)	
H14D	0.4277	0.3875	0.2295	0.038*	
H14E	0.4804	0.2273	0.2926	0.038*	
H14F	0.4942	0.3711	0.3219	0.038*	
C15B	−0.3891 (2)	0.28448 (19)	1.04042 (12)	0.0302 (4)	
H15D	−0.4787	0.3487	1.0709	0.045*	
H15E	−0.3195	0.2299	1.0850	0.045*	
H15F	−0.4159	0.2134	1.0149	0.045*	

### Atomic displacement parameters (Å^2^)

**Table:**

	*U*^11^	*U*^22^	*U*^33^	*U*^12^	*U*^13^	*U*^23^
O1A	0.0211 (6)	0.0212 (5)	0.0269 (6)	−0.0024 (4)	−0.0026 (5)	0.0003 (5)
O2A	0.0190 (6)	0.0234 (5)	0.0185 (5)	−0.0032 (4)	−0.0011 (4)	−0.0030 (4)
O3A	0.0277 (6)	0.0198 (5)	0.0190 (5)	−0.0045 (4)	−0.0032 (5)	−0.0024 (4)
C1A	0.0218 (8)	0.0164 (7)	0.0184 (7)	−0.0070 (6)	−0.0060 (6)	−0.0011 (5)
C2A	0.0189 (8)	0.0160 (7)	0.0230 (8)	−0.0052 (6)	−0.0072 (6)	0.0007 (6)
C3A	0.0181 (8)	0.0146 (6)	0.0219 (7)	−0.0067 (6)	−0.0022 (6)	−0.0029 (6)
C4A	0.0224 (8)	0.0178 (7)	0.0175 (7)	−0.0070 (6)	−0.0042 (6)	−0.0009 (5)
C5A	0.0205 (8)	0.0171 (7)	0.0213 (7)	−0.0072 (6)	−0.0054 (6)	−0.0007 (6)
C6A	0.0190 (8)	0.0135 (6)	0.0208 (7)	−0.0067 (5)	−0.0033 (6)	−0.0021 (5)
C7A	0.0180 (8)	0.0159 (7)	0.0224 (7)	−0.0071 (6)	−0.0038 (6)	−0.0023 (6)
C8A	0.0139 (7)	0.0188 (7)	0.0206 (7)	−0.0066 (5)	−0.0031 (6)	−0.0030 (6)
C9A	0.0162 (7)	0.0159 (7)	0.0246 (8)	−0.0044 (5)	−0.0030 (6)	−0.0030 (6)
C10A	0.0188 (8)	0.0187 (7)	0.0217 (7)	−0.0048 (6)	−0.0016 (6)	−0.0064 (6)
C11A	0.0159 (7)	0.0198 (7)	0.0183 (7)	−0.0061 (6)	−0.0049 (6)	−0.0021 (6)
C12A	0.0200 (8)	0.0132 (6)	0.0240 (8)	−0.0047 (6)	−0.0034 (6)	−0.0014 (6)
C13A	0.0158 (7)	0.0157 (7)	0.0241 (8)	−0.0048 (5)	−0.0021 (6)	−0.0058 (6)
C14A	0.0262 (9)	0.0313 (9)	0.0202 (8)	−0.0025 (7)	0.0004 (7)	−0.0024 (6)
C15A	0.0318 (9)	0.0270 (8)	0.0204 (8)	−0.0048 (7)	−0.0011 (7)	−0.0072 (6)
O1B	0.0300 (7)	0.0210 (5)	0.0235 (6)	0.0013 (5)	−0.0042 (5)	−0.0016 (4)
O2B	0.0181 (6)	0.0246 (5)	0.0178 (5)	−0.0045 (4)	−0.0018 (4)	−0.0023 (4)
O3B	0.0216 (6)	0.0232 (5)	0.0215 (6)	−0.0039 (4)	0.0014 (4)	−0.0033 (4)
C1B	0.0170 (7)	0.0168 (7)	0.0238 (8)	−0.0065 (6)	−0.0018 (6)	−0.0058 (6)
C2B	0.0158 (7)	0.0174 (7)	0.0216 (7)	−0.0019 (6)	−0.0072 (6)	−0.0044 (6)
C3B	0.0189 (8)	0.0139 (6)	0.0191 (7)	−0.0056 (5)	−0.0036 (6)	−0.0041 (5)
C4B	0.0162 (7)	0.0192 (7)	0.0220 (7)	−0.0056 (6)	−0.0008 (6)	−0.0059 (6)
C5B	0.0187 (8)	0.0154 (7)	0.0223 (7)	−0.0032 (6)	−0.0058 (6)	−0.0029 (6)
C6B	0.0197 (8)	0.0136 (6)	0.0202 (7)	−0.0046 (5)	−0.0029 (6)	−0.0038 (5)
C7B	0.0213 (8)	0.0153 (7)	0.0208 (7)	−0.0056 (6)	−0.0038 (6)	−0.0033 (6)
C8B	0.0198 (8)	0.0181 (7)	0.0172 (7)	−0.0061 (6)	−0.0036 (6)	−0.0025 (5)
C9B	0.0234 (8)	0.0150 (7)	0.0215 (7)	−0.0063 (6)	−0.0051 (6)	−0.0013 (6)
C10B	0.0235 (8)	0.0196 (7)	0.0198 (7)	−0.0101 (6)	−0.0040 (6)	0.0006 (6)
C11B	0.0175 (8)	0.0205 (7)	0.0177 (7)	−0.0044 (6)	−0.0060 (6)	−0.0033 (6)
C12B	0.0233 (8)	0.0146 (6)	0.0200 (7)	−0.0049 (6)	−0.0064 (6)	−0.0020 (5)
C13B	0.0221 (8)	0.0162 (7)	0.0179 (7)	−0.0075 (6)	−0.0060 (6)	−0.0002 (5)
C14B	0.0215 (8)	0.0314 (8)	0.0200 (8)	−0.0050 (7)	0.0012 (6)	−0.0050 (6)
C15B	0.0244 (9)	0.0311 (9)	0.0293 (9)	−0.0089 (7)	0.0048 (7)	0.0002 (7)

### Geometric parameters (Å, °)

**Table:**

O1A—C7A	1.2272 (17)	O1B—C7B	1.2257 (17)
O2A—C3A	1.3623 (17)	O2B—C3B	1.3550 (17)
O2A—C14A	1.4394 (18)	O2B—C14B	1.4336 (18)
O3A—C11A	1.3576 (17)	O3B—C11B	1.3599 (17)
O3A—C15A	1.4377 (17)	O3B—C15B	1.4343 (19)
C1A—C2A	1.374 (2)	C1B—C2B	1.382 (2)
C1A—C6A	1.399 (2)	C1B—C6B	1.402 (2)
C1A—H1A	0.93	C1B—H1B	0.93
C2A—C3A	1.393 (2)	C2B—C3B	1.396 (2)
C2A—H2A	0.93	C2B—H2B	0.93
C3A—C4A	1.399 (2)	C3B—C4B	1.398 (2)
C4A—C5A	1.383 (2)	C4B—C5B	1.389 (2)
C4A—H4A	0.93	C4B—H4B	0.93
C5A—C6A	1.398 (2)	C5B—C6B	1.396 (2)
C5A—H5A	0.93	C5B—H5B	0.93
C6A—C7A	1.4893 (19)	C6B—C7B	1.487 (2)
C7A—C8A	1.490 (2)	C7B—C8B	1.491 (2)
C8A—C9A	1.3930 (19)	C8B—C9B	1.392 (2)
C8A—C13A	1.405 (2)	C8B—C13B	1.4035 (19)
C9A—C10A	1.385 (2)	C9B—C10B	1.391 (2)
C9A—H9A	0.93	C9B—H9B	0.93
C10A—C11A	1.395 (2)	C10B—C11B	1.396 (2)
C10A—H10A	0.93	C10B—H10B	0.93
C11A—C12A	1.4012 (19)	C11B—C12B	1.397 (2)
C12A—C13A	1.377 (2)	C12B—C13B	1.378 (2)
C12A—H12A	0.93	C12B—H12B	0.93
C13A—H13A	0.93	C13B—H13B	0.93
C14A—H14A	0.96	C14B—H14D	0.96
C14A—H14B	0.96	C14B—H14E	0.96
C14A—H14C	0.96	C14B—H14F	0.96
C15A—H15A	0.96	C15B—H15D	0.96
C15A—H15B	0.96	C15B—H15E	0.96
C15A—H15C	0.96	C15B—H15F	0.96
			
C3A—O2A—C14A	117.70 (12)	C3B—O2B—C14B	117.78 (12)
C11A—O3A—C15A	117.33 (11)	C11B—O3B—C15B	117.90 (12)
C2A—C1A—C6A	121.10 (14)	C2B—C1B—C6B	120.88 (14)
C2A—C1A—H1A	119.5	C2B—C1B—H1B	119.6
C6A—C1A—H1A	119.5	C6B—C1B—H1B	119.6
C1A—C2A—C3A	120.14 (14)	C1B—C2B—C3B	120.21 (14)
C1A—C2A—H2A	119.9	C1B—C2B—H2B	119.9
C3A—C2A—H2A	119.9	C3B—C2B—H2B	119.9
O2A—C3A—C2A	115.76 (13)	O2B—C3B—C2B	115.56 (13)
O2A—C3A—C4A	124.33 (13)	O2B—C3B—C4B	124.67 (13)
C2A—C3A—C4A	119.90 (13)	C2B—C3B—C4B	119.76 (14)
C5A—C4A—C3A	119.20 (14)	C5B—C4B—C3B	119.40 (14)
C5A—C4A—H4A	120.4	C5B—C4B—H4B	120.3
C3A—C4A—H4A	120.4	C3B—C4B—H4B	120.3
C4A—C5A—C6A	121.50 (14)	C4B—C5B—C6B	121.46 (14)
C4A—C5A—H5A	119.2	C4B—C5B—H5B	119.3
C6A—C5A—H5A	119.2	C6B—C5B—H5B	119.3
C5A—C6A—C1A	118.13 (13)	C5B—C6B—C1B	118.27 (14)
C5A—C6A—C7A	118.48 (13)	C5B—C6B—C7B	119.27 (13)
C1A—C6A—C7A	123.22 (13)	C1B—C6B—C7B	122.33 (13)
O1A—C7A—C6A	120.49 (13)	O1B—C7B—C6B	120.47 (13)
O1A—C7A—C8A	119.53 (13)	O1B—C7B—C8B	120.05 (13)
C6A—C7A—C8A	119.96 (12)	C6B—C7B—C8B	119.47 (12)
C9A—C8A—C13A	117.96 (14)	C9B—C8B—C13B	118.68 (13)
C9A—C8A—C7A	118.92 (12)	C9B—C8B—C7B	118.98 (13)
C13A—C8A—C7A	123.05 (12)	C13B—C8B—C7B	122.22 (14)
C10A—C9A—C8A	121.65 (13)	C10B—C9B—C8B	121.10 (13)
C10A—C9A—H9A	119.2	C10B—C9B—H9B	119.5
C8A—C9A—H9A	119.2	C8B—C9B—H9B	119.5
C9A—C10A—C11A	119.62 (13)	C9B—C10B—C11B	119.45 (14)
C9A—C10A—H10A	120.2	C9B—C10B—H10B	120.3
C11A—C10A—H10A	120.2	C11B—C10B—H10B	120.3
O3A—C11A—C10A	124.78 (13)	O3B—C11B—C10B	124.69 (14)
O3A—C11A—C12A	115.67 (12)	O3B—C11B—C12B	115.42 (12)
C10A—C11A—C12A	119.55 (14)	C10B—C11B—C12B	119.88 (13)
C13A—C12A—C11A	120.05 (13)	C13B—C12B—C11B	120.14 (13)
C13A—C12A—H12A	120.0	C13B—C12B—H12B	119.9
C11A—C12A—H12A	120.0	C11B—C12B—H12B	119.9
C12A—C13A—C8A	121.14 (13)	C12B—C13B—C8B	120.72 (14)
C12A—C13A—H13A	119.4	C12B—C13B—H13B	119.6
C8A—C13A—H13A	119.4	C8B—C13B—H13B	119.6
O2A—C14A—H14A	109.5	O2B—C14B—H14D	109.5
O2A—C14A—H14B	109.5	O2B—C14B—H14E	109.5
H14A—C14A—H14B	109.5	H14D—C14B—H14E	109.5
O2A—C14A—H14C	109.5	O2B—C14B—H14F	109.5
H14A—C14A—H14C	109.5	H14D—C14B—H14F	109.5
H14B—C14A—H14C	109.5	H14E—C14B—H14F	109.5
O3A—C15A—H15A	109.5	O3B—C15B—H15D	109.5
O3A—C15A—H15B	109.5	O3B—C15B—H15E	109.5
H15A—C15A—H15B	109.5	H15D—C15B—H15E	109.5
O3A—C15A—H15C	109.5	O3B—C15B—H15F	109.5
H15A—C15A—H15C	109.5	H15D—C15B—H15F	109.5
H15B—C15A—H15C	109.5	H15E—C15B—H15F	109.5
			
C6A—C1A—C2A—C3A	−0.9 (2)	C6B—C1B—C2B—C3B	−0.8 (2)
C14A—O2A—C3A—C2A	−177.69 (14)	C14B—O2B—C3B—C2B	−178.89 (13)
C14A—O2A—C3A—C4A	3.1 (2)	C14B—O2B—C3B—C4B	2.0 (2)
C1A—C2A—C3A—O2A	−177.55 (13)	C1B—C2B—C3B—O2B	−178.01 (12)
C1A—C2A—C3A—C4A	1.7 (2)	C1B—C2B—C3B—C4B	1.1 (2)
O2A—C3A—C4A—C5A	178.31 (13)	O2B—C3B—C4B—C5B	178.98 (13)
C2A—C3A—C4A—C5A	−0.9 (2)	C2B—C3B—C4B—C5B	−0.1 (2)
C3A—C4A—C5A—C6A	−0.7 (2)	C3B—C4B—C5B—C6B	−1.4 (2)
C4A—C5A—C6A—C1A	1.5 (2)	C4B—C5B—C6B—C1B	1.7 (2)
C4A—C5A—C6A—C7A	176.83 (14)	C4B—C5B—C6B—C7B	177.53 (13)
C2A—C1A—C6A—C5A	−0.6 (2)	C2B—C1B—C6B—C5B	−0.6 (2)
C2A—C1A—C6A—C7A	−175.76 (14)	C2B—C1B—C6B—C7B	−176.32 (13)
C5A—C6A—C7A—O1A	−19.5 (2)	C5B—C6B—C7B—O1B	−24.1 (2)
C1A—C6A—C7A—O1A	155.62 (15)	C1B—C6B—C7B—O1B	151.59 (15)
C5A—C6A—C7A—C8A	162.10 (14)	C5B—C6B—C7B—C8B	156.77 (14)
C1A—C6A—C7A—C8A	−22.8 (2)	C1B—C6B—C7B—C8B	−27.6 (2)
O1A—C7A—C8A—C9A	−32.7 (2)	O1B—C7B—C8B—C9B	−32.4 (2)
C6A—C7A—C8A—C9A	145.72 (15)	C6B—C7B—C8B—C9B	146.71 (15)
O1A—C7A—C8A—C13A	144.16 (16)	O1B—C7B—C8B—C13B	143.46 (16)
C6A—C7A—C8A—C13A	−37.4 (2)	C6B—C7B—C8B—C13B	−37.4 (2)
C13A—C8A—C9A—C10A	1.4 (2)	C13B—C8B—C9B—C10B	0.8 (2)
C7A—C8A—C9A—C10A	178.38 (14)	C7B—C8B—C9B—C10B	176.84 (15)
C8A—C9A—C10A—C11A	−1.7 (2)	C8B—C9B—C10B—C11B	−1.3 (2)
C15A—O3A—C11A—C10A	−1.6 (2)	C15B—O3B—C11B—C10B	0.6 (2)
C15A—O3A—C11A—C12A	177.99 (14)	C15B—O3B—C11B—C12B	−179.56 (14)
C9A—C10A—C11A—O3A	179.83 (14)	C9B—C10B—C11B—O3B	180.00 (15)
C9A—C10A—C11A—C12A	0.2 (2)	C9B—C10B—C11B—C12B	0.1 (2)
O3A—C11A—C12A—C13A	−178.09 (14)	O3B—C11B—C12B—C13B	−178.39 (14)
C10A—C11A—C12A—C13A	1.6 (2)	C10B—C11B—C12B—C13B	1.5 (2)
C11A—C12A—C13A—C8A	−1.9 (2)	C11B—C12B—C13B—C8B	−2.0 (2)
C9A—C8A—C13A—C12A	0.4 (2)	C9B—C8B—C13B—C12B	0.8 (2)
C7A—C8A—C13A—C12A	−176.43 (15)	C7B—C8B—C13B—C12B	−175.07 (15)

### Hydrogen-bond geometry (Å, °)

**Table:**

*D*—H···*A*	*D*—H	H···*A*	*D*···*A*	*D*—H···*A*
C14B—H14E···O1B^i^	0.96	2.59	3.446 (2)	149
C9B—H9B···Cg1^ii^	0.93	2.84	3.5252 (17)	132
C12B—H12B···Cg1^iii^	0.93	2.78	3.5223 (16)	137
C4B—H4B···Cg2^iv^	0.93	2.88	3.6301 (18)	138
C9A—H9A···Cg3^iii^	0.93	2.92	3.5723 (16)	128
C12A—H12A···Cg3^ii^	0.93	2.88	3.5651 (16)	132
C4A—H4A···Cg4^v^	0.93	2.90	3.6376 (17)	138

Symmetry codes: (i) −*x*+1, −*y*, −*z*+1; (ii) −*x*, −*y*, −*z*+1; (iii) −*x*, −*y*+1, −*z*+1; (iv) *x*+1, *y*, *z*; (v) *x*, *y*, *z*−1.
